# Investigation of different ML approaches in classification of emotions induced by acute stress

**DOI:** 10.1016/j.heliyon.2023.e23611

**Published:** 2023-12-11

**Authors:** Heba Sourkatti, Kati Pettersson, Bart van der Sanden, Mikko Lindholm, Johan Plomp, Ilmari Määttänen, Pentti Henttonen, Johanna Närväinen

**Affiliations:** a VTT Technical Research Center of Finland, Tekniikantie 1, 02150 Espoo, Finland; b University of Helsinki, Department of Psychology and Logopedics, Faculty of Medicine, P.O. Box 63, 00014 University of Helsinki, Finland; c Eindhoven University of Technology, Electrical Engineering, Netherlands

**Keywords:** Affective computing, Emotion, Machine learning, Psychophysiology, Behavioral trait

## Abstract

**Background:**

Machine learning is becoming a common tool in monitoring emotion. However, methodological studies of the processing pipeline are scarce, especially ones using subjective appraisals as ground truth.

**New method:**

A novel protocol was used to induce cognitive load and physical discomfort, and emotional dimensions (arousal, valence, and dominance) were reported after each task. The performance of five common ML models with a versatile set of features (physiological features, task performance data, and personality trait) was compared in binary classification of subjectively assessed emotions.

**Results:**

The psychophysiological responses proved the protocol was successful in changing the mental state from baseline, also the cognitive and physical tasks were different. The optimization and performance of ML models used for emotion detection were evaluated. Additionally, methods to account for imbalanced classes were applied and shown to improve the classification performance.

**Comparison with existing method(s):**

Classification of human emotional states often assumes the states are determined by the stimuli. However, individual appraisals vary. None of the past studies have classified subjective emotional dimensions with a set of features including biosignals, personality and behavior.

**Conclusion:**

Our data represent a typical setup in affective computing utilizing psychophysiological monitoring: N is low compared to number of features, inter-individual variability is high, and class imbalance cannot be avoided. Our observations are a) if possible, include features representing physiology, behavior and personality, b) use simple models and limited number of features to improve interpretability, c) address the possible imbalance, d) if the data size allows, use nested cross-validation.

## Highlights

• Integrating biosignals, personal appraisals & behavioral data for ML-based emotional state classification.
• ML was used to classify the subjective emotional state following everyday-relevant tasks.
• A dive into practical considerations for ML in emotional classification.

## 1 Introduction

Human cognitive capacity affects behavior, thinking, and performance. Affective computing aims to provide information on the cognitive states such as emotions, cognitive load, or acute stress that affect the cognitive capacity [56]. The common methods used in affective computing include speech analysis, face reading, and behavioral monitoring; often integrating the information collected from several sources [58]. However, the cognitive state is reflected also in biosignals of the body and brain: the human body adjusts its nervous system to respond to changes in everyday situations and these adjustments cause changes that can be detected with different biosensors (e.g., [64]). A field where this approach has been applied for a long time is consumer behavior research, and the methodology and information derived from different measurements was recently reviewed in [3].

As wearable technology and data connectivity has evolved, biosignal monitoring and cognitive state assessment are becoming realistic in daily life. In field conditions, the selection of the measurement devices and fidelity of data connectivity is always a compromise between cost, complexity and restrictiveness of the measurement setup versus device specifications (e.g. number of channels, sampling rate, signal-to-noise) and probability of artifacts and lost data. Consequently, some of the data quality will be sacrificed. However, this can be at least partly compensated by using several sources of information [66]. Monitoring and detection of mental states promise benefits, such as improved stress management and better productivity and wellbeing, to both the individual user as well as for employers of communities, but arises a set of issues related to ethics and legislation, which in turn dictate what measurement methods and data usage are possible in real-life contexts [12], [61].

The selection of signals sources and features, as well as the choice of the model, for interpreting the cognitive state from these noisy and highly individual signal dynamics is not trivial and tends to be very context-specific. For robust automated emotion and stress detection, Machine Learning (ML) algorithms have been proposed and applied with promising results (e.g. [8], [66]). However, the ML approach requires well-defined ground truth for classification otherwise the outcome is erroneous [10]. Obtaining the ground truth can be a great challenge even in laboratory studies on cognitive states, not to mention cognitive state monitoring in the wild. Therefore, the field protocols eliciting affective states requires careful testing in the laboratory to be sure that the ground truth is valid and the selected ML approach robust and accurate.

In this work, we examine the emotional state related to real world-like stressful and cognitively demanding situations/conditions and further explore robust ML approach to tackle the challenges related to real-life situations such as noisy data and small data sets, using a controlled lab setting. A novel cognitive load and stress protocol is used to induce different mental states while monitoring biosignals (HR, HRV, EDA, EEG, and eye blinks). The tasks are not designed to induce any specific emotion, and the target of classification is the *subjectively* reported post-task emotional state: valence, arousal, and dominance. The classification of the emotional state uses a versatile set of input parameters (physiological features, task performance data, and sisu personality trait), in order to get a comprehensive insight into the role of different features in the human cognitive state.

As outcome, we demonstrate the optimization and performance of different ML algorithms for this type of challenge. We discuss the choice of the model, model accuracy and stability as well as practical problems and limitations in applying ML in experimental human biosignal data.

In the following chapters, the background to this multidisciplinary approach is summarized.

### 1.1 Psychology

There are two main approaches to define emotions. The first approach is to discuss specific, named emotions like joy, anger, fear etc. Typically these are divided into basic and other emotions, where the basic emotions are defined to be discrete (from each other), have specific neural basis and behavioral outcomes, and represent quite primitive fast responses to a stimulus. There are several emotion categorization models which have been summarized and applied in [78]. Another way to approach the emotional state is to look at the affect representation and recognition in valence–arousal–dominance space. Valence indicates the axis of negative/bad vs. positive/good, arousal calm vs. excited/agitated, and dominance submissive/weak or powerful/strong. These are illustrated in 3D continuous space [9], [33], [44], and the specific emotions can be positioned in this space, e.g. rage has low valence, high arousal and often high dominance, while relaxed is positive valence, low arousal and neutral dominance. The same basic emotion (happy) may have very different arousal: content happy is different from exited happy.

Besides asking directly, the emotions, both specific and 3D space ratings, can be detected objectively. In classical affective computing, monitoring of behavior (facial expressions, natural speech, gestures, micro movements) and, in some cases, biosignals have been used. In addition, smartphone usage and data from embedded sensors can be utilized in detecting emotions, moods and other behavioral characteristics (a review by [57]). In sentiment analysis, text produced by a person is used to infer emotions, emotional state, or polarity of the writer's opinion on the topic [49]. The fundamental source for affective computing is [56], and the psychophysiological emotional responses, relevant to this paper, are discussed in detail in Chapter 1.2.

In experimental settings, the cognitive states studied are typically induced. Emotions are often initiated by showing pre-tested and classified photos, film clips, or recalling a personal memory of an emotional event with questionnaires (see reviews [8], [66]). Paradigms for stress and different types of cognitive load (working memory, executive control, acute stress) exist as well (e.g., [66], [73]). This pre-determined mental state is then considered as a ground truth in the data analysis and interpretation. However, inter-personal differences in how the paradigm succeeds are large, especially in emotion induction (e.g., [41], [8], [66]). This becomes obvious when the stimuli are more complex, such as demanding cognitive tasks or stressors. Another way to define the ground truth is to collect subjective appraisals of the intensity of the targeted cognitive/emotional state and use this information either to classify the state individually or take the intensity into the model as a co-variant. The subjective appraisals are sensitive to various biases: the skill of specifying one's cognitive and emotional state varies, social pressure and personal values and beliefs influence what is reported, and the limited capacity of the memory may emphasize the later parts of the experience [19], [51]. For quantitative rating of emotion amplitude or emotional state, the participants tend to use their individual scale, both in what is neutral or normal, and what is high or low. The aspects of subjective classification approach has been discussed in detail in e.g. [29] and [8].

Personality, as characterized by validated questionnaires such as those measuring the facets of the five factor model, attention and subjective experience and coping in positive and adverse situations are linked [6], [31], [27], [16]. Personality has been associated with well-being and physiological stress-reactivity [41], and personality and behavioral traits were recently shown to interact with self-reported emotions and affects, psychophysiological stress reactivity, and movement activity in an analysis of real-life measurement with stress sensors and mobile self-reporting [40]. Behavioral traits relevant for coping in work life can be probed in terms of resilience and grit. In Finnish culture, the courage to overcome all kinds of negative challenges is termed *sisu* [32]. Sisu is a cultural construct that defines a person as being able to carry on until reaching the target and coping with any situation to achieve the goal. Hence, sisu can be the key trait to success - or (a contributor to) stubbornness leading to burnout. Recently, we developed a sisu questionnaire and a sisu scale. Sisu has two subscales, beneficial and harmful sisu: beneficial sisu S1 helps an individual to carry on in challenging situations while harmful sisu S2 urges to continue trying, often alone, neglecting advice and other duties, in a rationally hopeless pursuit. Sisu was strongly associated with well-being among German students, in a recent conference paper ([21]; German translation) and the full paper on the larger Finnish sample has been submitted.

### 1.2 Psychophysiology

The cognitive state, including emotions but also states like stress, flow, attention/relaxation etc. is reflected in the autonomous nervous system (ANS) function, which again influences several physiological signals: heart rate (HR) and its variability (HRV), respiration rate (RR), electrodermal activity (EDA), and temperature. For a summary of these responses seen during induction of different specific emotions, the review by [30] is an excellent starting point. More recently, emotion detection methodology was reviewed in [17]. Instead focusing on specific emotions, the emotional state can be mapped in the three-dimensional space of arousal, valence, and dominance. It is apparent that in emotions the arousal is the main driver of the ANS responses and that in some cases, dominance plays a role as well. Generally, high arousal accelerates the ANS system increasing HR, breathing rate, and EDA and reducing high frequency content of HRV. However, if the dominance is low, such as in passive, helpless fear or sadness, the acceleration is inhibited [30]. The effects of (acute) stress and cognitive load are somewhat similar to high arousal emotions. The literature on the topic is vast and best approached with review articles [13]. Combining a set of parameters, different stress indices can be computed, e.g. in [1] an index based on temperature and heart dynamics was introduced. In [55] heart and eye parameters were used to classify different stress types (cognitive load vs. physical discomfort). While in [18] indices based on the temporal characteristic of biosignals were used to assess the balance of the ANS.

Attention and eye activity are closely linked, humans scan the environment with rapid saccadic eye movements and the information gathered during the fixations. Thus, the dynamics of the eye movements and blinks as well as pupillary responses are affected by both visual stimuli [71], [11] and cognitive status (e.g. cognitive load, vigilance, engagement) [43], [20], [52], [54], [42], [60]. Even though, the eye activity comprises valuable information on perception and cognitive state, it is rarely used in affective computing ([34]). In recent publications, especially the blink parameters such as duration, time between blinks, and blink rate, variability and dynamics of the blink rate have been reported to be reliable estimates of the cognitive state [52], [42], [55], [60].

Electroencephalography (EEG) is one of the few measurements of brain activity that is somewhat feasible in the field. Again, the literature on EEG, emotions, stress, and cognitive load is vast but in this Introduction the focus is on simple EEG parameters which can be measured with wearable EEG headsets using a limited number of channels. In neuromarketing context, inverse values of alpha band power have been assigned to visual attention (occipital), approach motivation (frontal asymmetry), assessment (frontal+central) [81], [28]. For cognitive load, a parameter termed Brainbeat (BB) was introduced by [23] as the ratio theta frontal Fz/alpha parietal Pz. The study confirmed that Brainbeat can be used to measure overall brain load and estimate cognitive overload, as it reflects both external and internal load. A recent review summarizes the use of EEG in quantifying various aspects of cognitive performance [25]. Beyond these simple interpretations based on alpha power, a range of more advanced methods have been proposed, based on e.g. neural networks [84] or transfer learning [35].

Neural correlates of specific emotions have been reported mainly in brain imaging and electrical stimulation studies [78], [62]. Even in EEG, which measures cortical brain activity with low spatial resolution, specific emotions are hard to recognize, especially with wearable EEG devices [59]. The ANS responses are dominated by the degree of arousal, affected also by dominance, and assessing valence from them is difficult [30], making ANS non-optimal for detecting specific emotions.

### 1.3 Machine learning in classification of emotions

Using ML to extract information from data, i.e. features such as biosignals and behavioral parameters, measured from humans has some specific challenges. Typically, the number of subjects is low compared to the number of features, and some form of feature selection must be done (e.g., [8]). The data is often imbalanced as well: the different states or cases are not equally presented. This has consequences on the model selection, model tuning and in estimating the model performance. These issues in the context of healthcare decision support systems were recently reviewed in [77]. In classification of human cognitive states these shortcomings are even more pronounced, as the data acquisition protocols tend to be tedious, resulting in number of participants typically in tens rather than in hundreds, and the balance of the data cannot be fully controlled; the participants' responses, appraisals and behaviors are individual.

Recently, an increasing number of studies have shown promising results using ML in classification of cognitive states. However, the comparison of these studies is difficult since the protocols vary considerably: state induction methods (tasks, videos etc.), the number of subjects, the biosignals and features utilized, the choice of classifiers, models, validation methods, and the classification basis (type of stimulus vs. subjective assessment), (e.g., review by [8]). Typically, the classification accuracy in these studies has been in the range of 60 – 80%, but much lower and higher rates have been reported. Model personalization has been found to improve the classification accuracy in physiological features based stress detection (e.g., [69], [76], [55]), but less attention has been paid to use personality traits or task-related subjective evaluations as input features in the ML models.

As defining the ground truth (i.e., classes) is one of the most important steps in ML-based classification, the most common approaches are presented here. First, the stimulus or task needs to be selected carefully and preferably pre-validated (e.g., emotional video clips and photos) to ensure the target states or emotions are induced with strong enough intensity. The classes are based either on the stimulus type/class, assuming all participants experience the targeted state, or on the subjective assessment of the state or emotion after each stimulus. Also, a combination of these has been used (e.g., [69]). In a recent article by [18], stress was induced with a cold pressor task and pre-defined neutral, pleasant and unpleasant film clips (N=26). The physiology was monitored by electrocardiogram (ECG) and EDA sensors, indices for temporal characteristics of sympatho-vagal balance were extracted, and the classification was done between rest and cold pressor task, and between pleasant (high valence) and unpleasant (low valence) emotional states. A support vector machine (SVM) approach with a recursive feature elimination was used as ML model and it was validated with Leave-One-Subject-Out (LOSO) procedure. The 73% classification accuracy for valence was achieved with only four features (two sympatho-vagal balance and two HRV spectral parameters). Chang et al. used pre-defined film clips to induce sadness, fear, and pleasure for 11 subjects [15]. Physiology was monitored by ECG, EDA, and blood volume pulse and the classification was done based on the film category using support machine regression. They reached 89% averaged accuracy for detecting three emotions.

As individual appraisals vary, classification has been based also on self-assessments of the emotion type and intensity, or by the reported emotional state in valence-arousal(-dominance) dimensions. [83] used a set of short movie clips, each one eliciting a single positive or negative emotion while collecting participant's physiological signals using EEG, as well as self-reported emotional states. The best classification accuracy of 92% was obtained by using SVM and linear discriminant analysis in feature selection (30 EEG features). [29] developed a database for emotion analysis using physiological signals by recording EEG and peripheral signals while participants watched music videos and rated their emotional states and appraisals (arousal, valence, dominance, liking, and familiarity) after each trial. The classification of high and low emotional states of arousal, valence, and liking was done by using Gaussian Naive Bayes (GNB). The valence was classified with 65% accuracy while arousal and liking were detected with 62% accuracy. In [86] visual stimuli (from international affective picture system) and auditory stimuli (from international affective digital sounds) were used to elicit emotions and the reported emotions were classified by decision tree and KNN models achieving 76% classification accuracy for both auditory and visual stimulus for the six basic emotions (excited, happy, neutral, sad, fearful, and disgusted). [72] modeled data from publicly available data sets in which the biosignal (EEG, HR + HRV, EDA, and face reading) responses during video-based emotion induction were tagged with valence, arousal, and liking ratings. Using deep learning methods for the data sets separately, they reached classification accuracies around 70 - 85% for the emotional state and liking classes, and around 45 - 60% for the four emotion type classes (the quadrants of the valence-arousal space, representing approximately happy/excited, annoying/angry, sad/bored, and calm/peaceful). When the data sets were combined and modeled as a whole, the classification accuracies dropped to 60 - 70% and ca. 40%, and were recovered slightly by introducing the transfer learning approach.

### 1.4 Machine learning models and validation

A variety of models has been used in classification of emotional states. Five often applied models and their key parameters are presented here:

1. Logistic Regression (LR) is a simple binary classification algorithm that learns how each of the features correlates with different targets. It is a generalized linear model where probabilities of an observation Y belonging to a class are estimated using a logistic function. The cost function is computed and minimized using an optimization algorithm. Additionally, a regularization term (with tuning parameter *λ*) is added to the cost function to avoid overfitting of the training data and help to generalize the model for new unseen data [7], [26]. LR is robust, quick to train and provides probability information. However, as an inherently linear model, it is not suitable for non-linear problems.

2. Support vector machines (SVM, [79]) is in a way similar to logistic regression but instead of predicting the classes based on a hard threshold it adds a margin between the classes boundaries. The decision boundary is selected to have the maximum possible distance (margin: perpendicular distance to the closest point) between the two classes. Hence, the SVM objective is to maximize the margin while softly penalize points that lie on the wrong side of the margin [45], [24]. SVM is a robust classifier but training is slow and the performance with overlapping classes is not good.

3. Random Forest (RF) is an ensemble of a large number of decision trees (DT; a non-parametric classifier that uses decision rules (if-then-else) to approximate the data curve). Multiple trees are built by using a bootstrapped data set and considering a random subset of variables at each step, resulting in a wide variety trees. This variety makes RFs more effective than individual decision trees. To classify a new sample with a Random Forest, the sample is classified by all DTs and the class with the majority votes will be the final classification result. This approach is known as bagging (i.e., bootstrap aggregate) [85], [5]. RF has good predictive power but is resource consuming and prone to overfitting.

4. K Nearest Neighbors (KNN) is a non-parametric model and one of the simplest yet effective classification algorithms. KNN clusters labeled training data in the future space. Further, to classify a new data sample with an unknown class, the K nearest neighbors to the new data sample are picked, and the class with the most votes by the selected neighbors is assigned to the new data sample class. The selection of the number of neighbors (K) is very crucial: low values can lead to outliers and noisy data, whereas too large values may neglect one class altogether, especially in unbalanced data sets. The selection of the distance measure is important, most common are Euclidean distance, Manhattan distance and Mahalanobis distance [24], [26]. KNN provides interpretable results only with a small number of features [46].

5. Gaussian Naive Bayes (GNB) is a type of Naive Bayes classifier based on the Bayes' theorem. Unlike the previous four discriminative classifiers, which only minimize the number of misclassification, GNB is a generative classifier, resulting in a probabilistic model. It assumes that the input data have a normal distribution and each feature is independent of any other feature values [50]. GNB has proven to be a robust classifier, especially with small training data, but often lacks power as an estimator. Also Cauchy Naive Bayes classifier has been used for emotion recognition [67].

The data collected in human behavior and health context is often highly imbalanced i.e. the populations of samples classes are not equal. Some models have built-in options (e.g., class_weight= balanced) for addressing this imbalance but there are models specifically designed for the task. The AdaBoost classifier is based on a combination of many weak learners (i.e., Decision Trees with a maximum depth of 1); each weak classifier is adapted according to the previous classifier mistake by adjusting the weights of misclassified cases [37]. Random Under Sampling (RUS) is a sampling technique used for an imbalanced data set to under sample the class with higher samples number (majority class) by randomly picking samples for each class individually. RUSBoost [68] is a classifier intended for learners built over imbalanced data. It is a hybrid of a sampling and a boosting algorithm, which combines specifically the random-under sampling technique and the AdaBoost classifier. Table 1 summarize the pros and cons of the above mentioned ML models.

**Table 1:** Comparison of key characteristics of the machine learning models.

**Model**	**Type**	**Pros**	**Cons**
**LR**	Linear Classifier	Simple and interpretable, works well with linearly separable data	May not perform well with complex or nonlinear relationships
**SVM**	Linear and Nonlinear Classifier	Powerful and effective with high-dimensional data, can handle nonlinear relationships	Computationally expensive, requires careful selection of hyperparameters
**RF**	Ensemble Method	Robust to overfitting, handles missing data well	Can be difficult to interpret, may not perform well with high-dimensional data
**KNN**	Nonparametric Classifier	Simple and easy to implement, can handle both classification and regression	Sensitive to distance metric, may not perform well with high-dimensional data
**GNB**	Probabilistic Classifier	Simple and computationally efficient, works well with small datasets	May not perform well with correlated features or require more data to achieve good performance
**AdaBoost**	Ensemble Method	Powerful and can be used for both classification and regression	Sensitive to noisy data and outliers
**RUSBoost**	Ensemble Method	Can handle class imbalance well and reduce the risk of overfitting	May not perform well with noisy data or in the presence of strong class overlap

**Figure 1:**
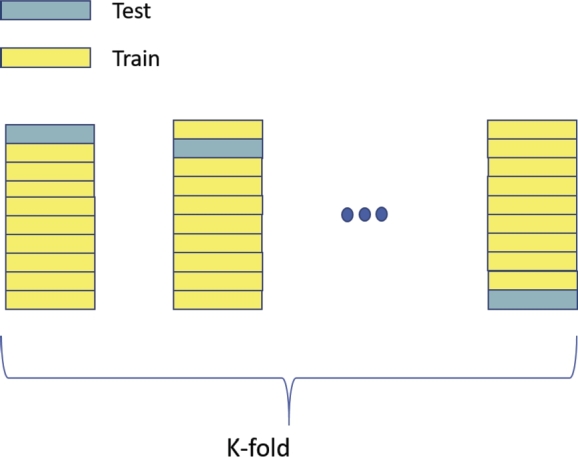
Leave-One-Out cross validation.

**Figure 2:**
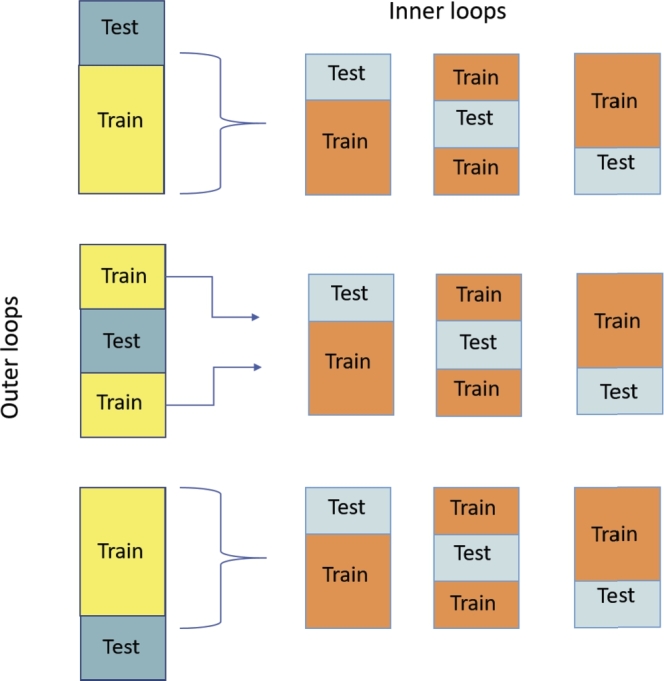
Nested cross validation.

The performance and generalizability of ML models are tested using cross-validation (CV) methods [75]. K-fold cross-validation randomly splits the data set into K different subsets, where each subset is used as a validation set while the remaining K-1 sets are used to train the model. Another variation of K-fold CV is when the K splits are created with the same percentage of samples for each class as the original data set, known as Stratified K-fold CV. A particular case of the K-fold CV is Leave-One-Out (LOO) CV when K equals the number of samples, and hence the model is tested once over each sample (Fig. 1). The CV can be done also in a nested manner by repeatedly splitting the data into multiple training and test sets (see Fig. 2). The nested CV seems to introduce less bias than K-fold CV, especially LOO. A review of cross-validation procedures was provided by [80], and the bias and variance in cross-validation are discussed also in [14]. Furthermore, [63] presents a comprehensive summary of the various validation techniques that are commonly utilized in studies on emotion recognition.

ML classification model performance can be evaluated using a number of metrics. Accuracy is the ratio of correctly classified cases and total number of cases, and describes the overall performance. Precision is a class-wise measure and reports the ratio of the number of true cases in a class and number of cases classified to that class. Recall is the ratio of number of correctly classified cases in a class and the total number of cases that should have been classified to a class. F1-score is the harmonic mean of precision and recall for each class. In [77] it is remarked that precision (positive prediction value in classification of more than two classes) and consequently F1-value are not clean indicators of the model performance since the balance of the cases in different classes influences precision. Shapley Additive Explanations (SHAP) values are a way to evaluate the individual predictions of a model. The magnitude of a SHAP value reflects the importance of a feature. In a SHAP summary plot, SHAP values are shown for each feature and, within a feature, for each subject or data instance. The actual values of the features are typically indicated by color, which allows investigation of the direction of the effect. For a more detailed description of Shapley values and SHAPs please see [46] and [38].

## 2 Experimental

### 2.1 Participants, protocol and measurements

A controlled lab setting was built to collect the physiological and emotional data from 26 right-handed adults (8 male and 18 female), aged between 19 and 39 (μ=24,σ=4.13). This sample size is typical for studies on human psychophysiology and affective computing (see e.g., [8], [30]). All subjects were healthy with no record of cardiac disorders or depression and no current consumption of medication that might affect the ANS. As the study protocol was in Finnish, native Finnish was required. Prior to the experiment, each participant filled out the sisu questionnaire [22] to compute their sisu scores for beneficial and harmful sisu (features sisu_S1 and sisu_S2, respectively). The participants were instructed to maintain their normal lifestyle, avoid any atypical activities during the 24 hours before the study visit, and ensure they do not arrive hungry nor very full. All visits were hosted by the same researcher and the experiments were conducted one participant at a time. All participants gave their informed consent in all of the studies. The participants were also informed that they could stop their participation at any given time and their participation was completely voluntary. The study proposal was evaluated by the Ethics Committee in the Humanities and Social and Behavioral Sciences of the University of Helsinki.

Throughout the experiment, the physiological signals were recorded using a 64-channel EEG system (Bittium NeuroOne, Bittium, Finland). Additionally, two bipolar channels were allocated for electrooculogram (EOG) measurement, one bipolar channel for ECG to measure HR and HRV, and one bipolar channel for EDA. For the latter, both electrodes were placed on the index and middle fingers of the left hand. The ECG electrodes were positioned below the left collarbone and on the right lower back. EOG was measured between the electrodes placed above and beneath the left eye (vertical) and the outer corners of the eyes (horizontal). The blinks were estimated from the vertical EOG signal. For EEG, the 10 - 20 electrode placements system was used. All signals were acquired at 1000 Hz with 0.16 Hz high-pass and 7 kHz low-pass analog filters.

There were five tasks in the protocol. For more a detailed description please see [39] for tasks 1, 2, 3, and 5, and [73] for MAST:

1. **Anagram** puzzles that require the individual to rearrange the letters of six-letter words into new words. Unbeknown to the participant, the task included anagrams of different difficulties, including impossible anagrams. Task instructions: Try your best but you can proceed to the next word by pressing enter.
2. Verbal **problem** puzzles 2 – 4 multiple choice answers. A translated example of an easy puzzle: “Let's assume all mermaids like ice cream. Anna is a mermaid. Does Anna like ice cream?” a) Yes, b) No, c) I cannot deduce from the given information. Some of the puzzles were easy, some somewhat harder and some (unbeknown to the participant) impossible to solve. Participants were promised more “points” from more difficult puzzles. Task instructions: Try your best but you can proceed to the next problem by pressing enter.
3. **Cold** water challenge that assesses the individual's pain threshold and tolerance when he/she immerses his/her hand in a bucket of icy water. Task instructions: Try to keep your hand wrist-deep for as long as you can. As soon as you start to feel discomfort, keep reporting the level of the pain you experience, on a scale of 0 – 10.
4. Maastricht Acute Stress Test (**MAST**) that alternates between hand immersion in cold water and mental arithmetic task [73].
5. Boring **video** where subjects watch an eventless video (as long as they desire) in order to answer some questions regarding the video.

**Figure 3:**
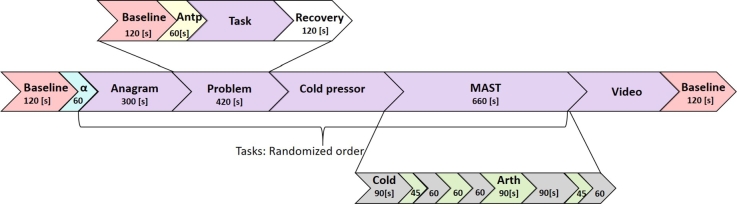
The experimental protocol. The substructure shown on the first line is repeated in each task. Only the first three tasks are discussed in this paper.

The experiment protocol is illustrated in Fig. 3, started with 120 [s] baseline measurement while participants were instructed to relax. After baseline, they were guided to keep their eyes closed for 60 [s] to induce alpha oscillations used in the assessment of individual alpha frequency (IAF). There were five tasks in the protocol. The MAST and boring video task results are not presented in this paper. All tasks and subjective evaluation forms were presented and logged using Presentation software (Neurobehavioral Systems, Inc, Berkeley, USA).

All tasks were structured as follows:

1. 120 [s] baseline.
2. Pre-task emotional rating questionnaire.
3. 60 [s] anticipation period after being briefed about the forthcoming task.
4. The task.
5. 120 [s] recovery period.
6. Post-task emotional rating questionnaire.

The individuals behaviors and emotional states were measured via pre- and post-task questionnaires. The emotional state was recorded in terms of valence, arousal, and dominance using self-assessment manikin [47]. The enthusiasm, tediousness, self-perceived performance, and performance compared to peers were asked, relating to the task at hand. All these ratings were collected using a discrete 1 – 9 scale. In the modeling, post-task ratings for enthusiasm (feature enthusiasm1) and performance compared to peers (feature perfpeers1) were used. The pain experienced during the cold immersion task was acquired on a scale of 0 – 10, and the maximum pain rating was used in the data analysis. The anagrams and problems included, among other tasks, insolvable subtasks, in order to measure the tendency to get stuck, indicative of high harmful sisu. The time spent on the impossible tasks was recorded (feature tDUR).

### 2.2 Signal analysis

All biosignal data analysis was carried out in Matlab (MathWorks, Natick MA, USA). Electrodes T9 and T10 were used as a linked mastoid reference. The EEG data were filtered with a bandpass of 1 – 40 Hz, eye movement artifacts were removed by regression and the signal was downsampled to 200 Hz. The EEG data from each task were Fourier transformed to yield spectra. First, IAFs were extracted from eyes-closed condition for each subject and the individual alpha band range was set to IAF ± 2 Hz and theta range 4 – 8 Hz. The Brainbeat parameter was computed as the ratio theta(Fz)/alpha(Pz) [23].

ECG data was first bandpass-filtered (3 – 30 Hz), artefacts were cleaned and then the data were squared for ECG peak detection, using a standard QRS Pan-Tompkins algorithm (HRV Tool in Matlab; [82]). From the resulting beat-to-beat interval time series, the HR and the root mean square of successive differences (RMSSD; a commonly used, robust parameter reflecting HRV) were computed. The EDA data were analyzed using the Ledalab toolbox [4] with default parameter settings. The EDA amplitude was computed by utilizing the phasic data. After successfully separating the GSR constituents, the amplitude of the phasic data was time-averaged per (sub)task. This average EDA amplitude provides an indication of both the number of EDA peaks and the magnitude of those peaks. The blinks were extracted from the EOG signal with an automated algorithm [53] and the average time between blinks (TBB) was extracted for each task. HR, RMSSD, EDA, BB, and TBB were derived using a 15 s moving window and then averaged over the task. For each subject, these mean values of the biosignal parameters during the tasks were normalized to the mean of the corresponding parameter measured during the baseline periods at the beginning and at the end of the protocol. For comparing the responses seen in different tasks, a pairwise, two-way t-test was used. As the Anagram and Problem tasks induced similar physiological responses and average emotion ratings, they were considered a single task with 52 (i.e. 2 x 26) samples.

### 2.3 ML classification of the subjective emotional state

The biosignal responses described above were used as ML features together with behavioral (sisu scores) and task-related parameters, This resulted in a total of 10 features that were used as inputs for the ML model. The full feature list is shown in Table 2. As discussed in the Introduction, the number of features available from biosignal measurements is very high compared to the number of subjects. Therefore feature selection must be done. Rather than including automated feature selection into the models, one feature was chosen to represent each biosignal. This choice was made to simplify the interpretation of the feature importance in the models.

**Table 2:**
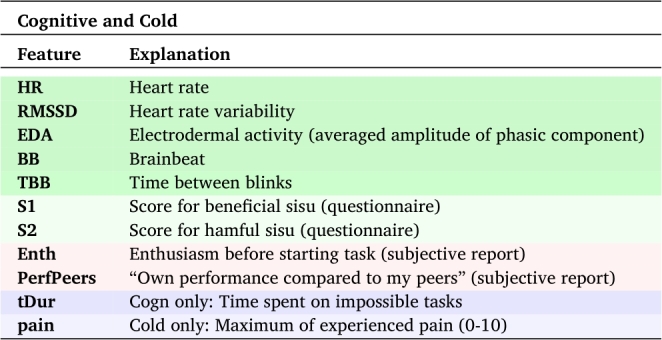
Features used in classification of arousal, valence, and dominance in Cognitive and Cold tasks.

The targets of classification were the three dimensions of emotion: arousal, valence, and dominance reported subjectively after each task on scale 1, 2,... 9. Values of 6, 7, 8, and 9 represented class “high” and the rest class “low”.

Since our data has a varying scale, all features were standardized to have a zero mean and scaled to unit variance independently (i.e., be normally distributed). This preprocessing step is required for many ML algorithms as they assume independent and identically distributed data. Also, features with differing scales fitted to an ML model would result in wrong contributions by adding more weight for the features with higher scales.

The flow of modeling efforts was result-driven and is described in more detail in the Results section. In brief, we first run the five models (LR, RF, SVM, KNN, and GNB) with standard settings for all tasks. We then tested two cross-validation techniques (LOOCV and nested CV) on arousal classification in the cognitive task. Finally, we used models considering the population balance for the imbalanced data sets (balanced RF and LR, GNB, RUSBoost, and balanced ADABoost). The model settings and hyperparameters are given in Appendix A. The performance of the classification was assessed by accuracy and standard deviation (CV tests) and precision, recall, F1-scores, and SHAP values. All ML modeling was run using Scikit-learn and imbalanced-learn libraries.

## 3 Results and discussion

### 3.1 Psychophysiological and subjective responses

**Figure 4:**
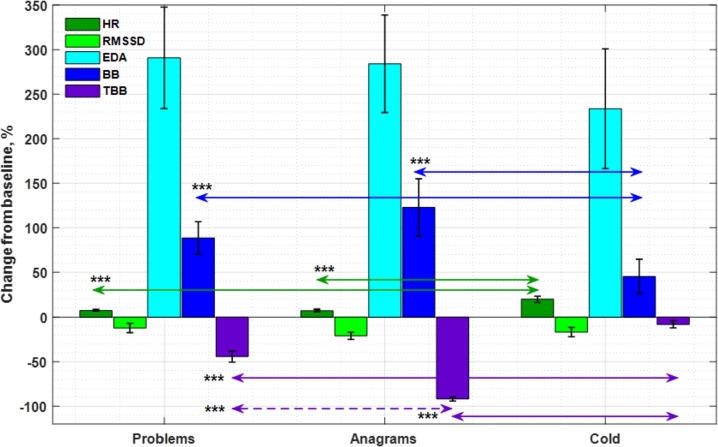
Relative responses in heart rate (HR), heart rate variability (RMSSD), skin conductivity (EDA), Brainbeat (BB), and blink interval (TBB), presented as group level averages with standard errors. The solid line arrows present statistically significant (n=26, pairwise t-test), differences between the two cognitive tasks and the Cold task. The only statistically significant difference between Problems and Anagrams task, seen for TBB, is marked with a dashed arrow. All shown differences had p < 0.01.

The average of the relative responses, as compared to the baseline, in Problem, Anagram, and Cold tests are shown in Fig. 4. The biosignal responses during tasks show the emotion induction was successful, the tasks increase arousal: HR is elevated and HRV decreases. EDA is much higher than at rest, and the Brainbeat amplitude is increased as well, indicating increased cognitive load while the time between blinks is decreased. When the cognitive tasks, Problems and Anagrams, were compared to the Cold task, some statistically significant differences were observed. HR in both Problem and Anagram tasks was lower than in Cold (p-values 5.5*$10^{-4}$ and 6.8*$10^{-4}$, respectively) while BB decreased in Cold compared to Problems and Anagrams (p-values 0.0056 and 0.0014, respectively). The rate of blinking in cognitive tasks was higher (lower TBB) than in Cold (p = 1.2*$10^{-4}$ for Problems and p = 2.6*$10^{-13}$ for Anagrams). This is in line with earlier studies on the effect of acute stress and cognitive load [52], [55]. The psychophysiological responses in the MAST task were recently published in [55]. The responses seen in heart dynamics and blink parameters in the two MAST phases (cold immersion and mental arithmetics) were even larger than in the tasks used in this paper. The difference is not unexpected, as MAST is a designed and validated stress induction paradigm and shown to arouse the ANS system and brain [70], [65]. However, Anagrams and Problems represent a more typical office work challenge than the MAST cognitive task with time-pressured counting and immediate penalty for mistakes.

In most parameters, there were no statistically significant differences in responses between Problem and Anagram tasks but both of these differed from Cold. In EDA, the responses were large, but high inter-individual variation is likely to obscure the possible differences. TBB was significantly shorter in Anagrams than in Problems (p = $8.4⁎10^{-9}$) indicating that the Anagram task could have been cognitively more demanding than the Problem task (e.g., [52], [55]). However, the visual stimulus and performance strategy have been most likely different in these tasks and these factors may affect the blinking frequencies (e.g., [71]). For instance, Anagrams may have required more visual effort forming candidate words for Anagrams while Problems require less visual imaginary – however this may be very individual (e.g., [52]). There was also a nearly significant trend in HRV (p = 0.076) and BB (p = 0.062): BB appears to be higher and HRV lower in Anagrams than in Problems, suggesting the Anagrams may have induced more cognitive load and even stress.

**Figure 5:**
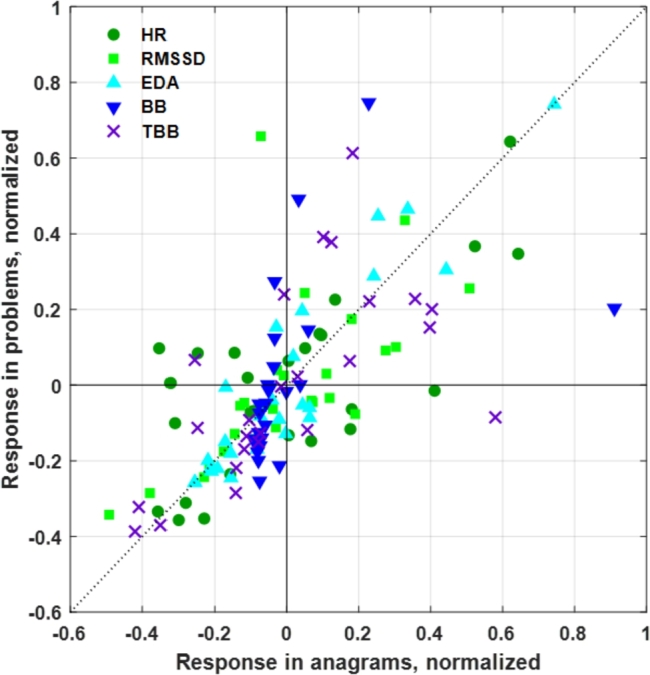
Normalized individual physiological responses in Anagram and Problem tasks. Each marker represents one participant.

Despite these differences, the two cognitive tasks seem to present quite similar mental states. To verify this assumption, the normalized individual responses were compared. The scatter plot is shown in Fig. 5. The inter-individual variation was high, yet for all parameters, the individual responses seen in the two tasks were similar, falling close to the line of unity in the graph. Based on this, to simplify the ML modeling part, we combine the Anagram and Problem task data into a task called Cognitive task, consisting of 52 samples e.g. two from each participant.

The three target vectors (post-task subjective ratings of arousal, valence and dominance) were binarized as high or low-class resulting in data sets shown in Table 3. The upper limit of the “low” category was set to 5 and this was fixed for each target. This results in a rather unbalanced population in some cases, particularly valence and dominance in Cold task. The other possibility would have been to treat each category independently and set the threshold to the mean of the given ratings. However, this would have broken the connection between the reported emotion direction (e.g. peaceful vs. excited for arousal, with value 5 being in the exact middle of these endpoints) and the corresponding data set. Further, the subjective ratings were integers from 1-9, a median-based split cannot ensure well-balanced populations in all cases. Similar choices about high and low state categories, and resulting imbalanced data were discussed by [29].

**Table 3:** Number of samples per class in cognitive (Cog) and cold pressor (Col) tasks and classification of valence (Val), arousal (Aro) and dominance (Dom).

**Class**∖**Emotion**	**Cog**	**Cog**	**Cog**	**Col**	**Col**	**Col**
	**Val**	**Aro**	**Dom**	**Val**	**Aro**	**Dom**
**low**	22	29	18	3	20	2
**high**	30	23	34	23	6	24

The correlations between ratings of arousal, valence and dominance, and the normalized features (Table 2) were checked. As the research question is based on emotional ratings within the same task (instead of ratings after different tasks designed to induce different emotional states), strong correlations between single parameter pairs were not expected. Yet there were some statistically significant correlations: in cognitive task, arousal with PerfPeers (R = 0.48), valence with RMSSD (R = −0.33) and TBB (R = −0.42), and dominance with enthusiasm (R = 0.36); and in Cold task only between valence and beneficial sisu S1 (R = 0.42). However, finding significant correlations among numerous comparisons is typical and even if these associations are interesting, further discussion of these is not in the focus of this paper.

There were certain recruitment criteria for the participants: age (18-45 years), no diagnosed cardiac problems, no medication affecting the nervous system, and right-handedness. As the emotion classification was done for a group of healthy adults, the models me not describe other populations as well. The lateralization of the brain is connected to handedness which has influence on EEG. However, the channels used in the Brainbeat parameter are located bilaterally around the midline and are insensitive to lateralization. The other parameters derived are not influenced by handedness.

### 3.2 ML models: implementation and validation

We trained five ML models (LR, SVM, RF, KNN, and GNB) to predict the 3D emotions during the cognitive and cold task for the 26 subjects and ten features (i.e., five physiological and five behavioral features, see Table 2). The selected ML models are commonly used in literature for emotion prediction. Furthermore, it is recommended to choose simple models for a small data set to avoid the high number of parameters to be learnt by the model [77].

Typically a separate training and validation data set is required to evaluate ML algorithms. However, dividing our data into training and validation sets is inefficient, due to the small data size. The common practice with small data is to run LOOCV, where the model will be tested over each sample as a test fold and trained with the rest of the data. However, without separate sets for hyperparameter tuning and model evaluation, this leads to biased estimates of the models' performance [77] but can be avoided using the nested CV technique. We ran experiments with various CV selections for the classification of arousal in the cognitive and cold tasks to comprehensively explore the use of the CV technique in a small sample size. The results are shown in Table 4 for LOOCV and Table 5 for Nested CV.

**Table 4:** LOOCV estimators averaged results for predicting arousal post-task in cognitive (Cog) and cold pressor (Col) tasks.

**Model**	**Results**			
	**Cog**		**Col**	
	**Accuracy**	**Std**	**Accuracy**	**Std**
**Logistic Regression**	0.62	0.49	0.73	0.44
**Random Forest**	0.71	0.45	0.73	0.44
**Support Vector Machine**	0.63	0.48	0.77	0.42
**KNearestNeighbor**	0.61	0.49	0.73	0.44
**GaussianNB**	0.65	0.48	0.77	0.42

**Table 5:** Nested-CV estimators averaged results for predicting arousal post-task in cognitive (Cog) and cold pressor (Col) tasks.

**Model**	**Results**			
	**Cog**		**Col**	
	**Accuracy**	**Std**	**Accuracy**	**Std**
**Logistic Regression**	0.65	0.05	0.54	0.27
**Random Forest**	0.69	0.03	0.54	0.07
**Support Vector Machine**	0.65	0.01	0.50	0.22
**KNearestNeighbor**	0.65	0.04	0.66	0.15
**GaussianNB**	0.60	0.04	0.73	0.05

The reported results for LOOCV show high variance between the performance on different folds for these two data sets; this might be due to the test's deterministic nature, measuring only high or low emotion per sample while the training samples are continuously repeated over the folds. The variance was decreased by using a stratified 3-fold CV for inner and outer CV loops (Table 5), particularly for the Cognitive task arousal, which has more samples (52) and better balance between the classes than the Cold task arousal (26 samples). Furthermore, unlike the LOOCV, the stratified k-fold in the nested CV has an appropriate representation of the original data in terms of class distribution. Using training data that resembles the class balance in highly imbalanced class divisions data improves the reliability of the results [77]. Consequently, we selected the nested CV technique for the rest of the experiments, by which we ensure not mixing the samples used by the model for parameter tuning with the samples used for model evaluation.

**Figure 6:**
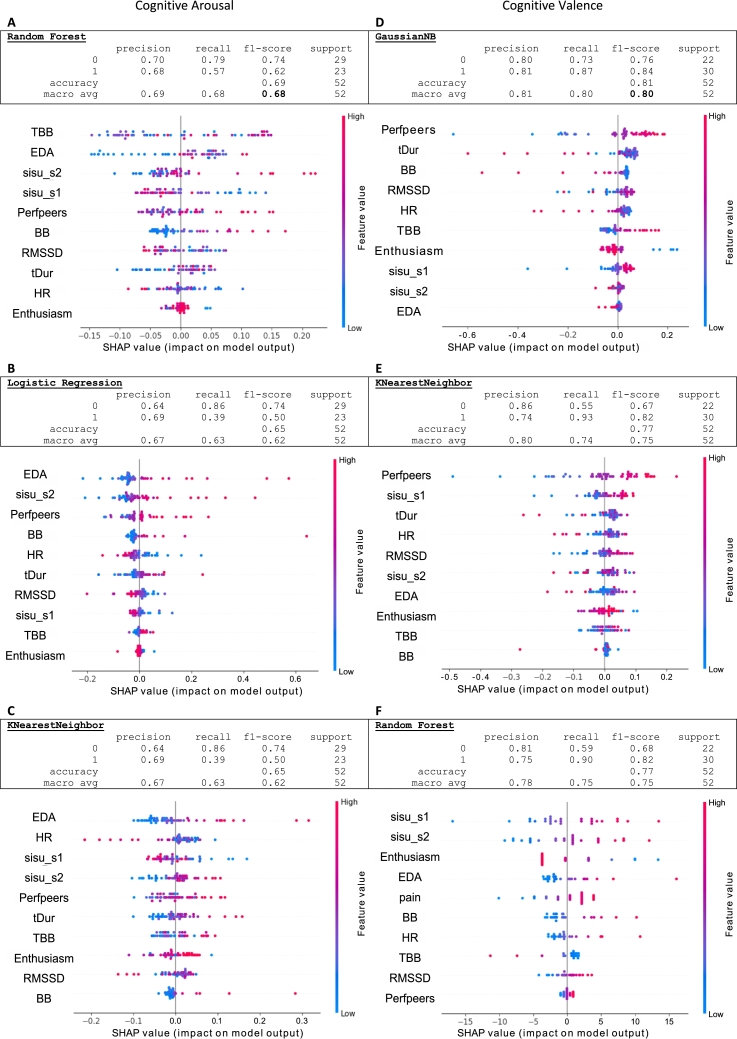
The best-performing models for classification arousal and valence in Cognitive task, for which the data were reasonably well balanced. The SHAP values indicate the importance of the feature in the model and SHAP values are colored to indicate the direction of the effect.

The full classification results are given in Appendix B. Fig. 6 shows the cognitive task classification reports and feature importances for the best models in classifying post-task arousal and valence. In the feature importance graphs, SHAP values are shown for each feature and within a feature, for each observation (subject). The farther the values are from the zero line, the greater their impact on the classification result. The color indicates the feature value of the data point and thereby reflects the direction of its effect.

For **arousal** classification in the cognitive task, RF reported the highest score with an average accuracy of 0.69 and an f1-score of 0.68, in which the model has f1 score 0.74 for predicted low arousal class and f1-score 0.62 for predicted high arousal class (Fig. 6 A). LR and KNN performed nearly as well (both with accuracy 0.65, f1 0.62, f1-low 0.74, and f1-high 0.50), (Fig. 6 B and C). In RF, time-between-blinks TBB and skin conductivity EDA are the most important features and both are related to high arousal. They are followed by harmful sisu S2 (related to high arousal) and beneficial sisu S1 (related to low arousal). The most important feature in LR and KNN is EDA, while TBB is not very important in either model.

For **valence** classification in the cognitive task, GNB was the best model (Fig. 6 D), detecting both classes with satisfying metrics: accuracy 81, f1-score 0.80, f1-low 0.76, f1-high 0.84 (e.g., compared to [8]). KNN and RF (Fig. 6 E and F) both performed at average accuracy of 0.77 and an f1-scores of 0.75 (f1-low 0.67 and 0.68, f1-high 0.82 and 0.82, respectively). The most important feature in all these models was the estimated performance compared to peers: better the estimation, higher the valence. In GNB and KNN, the time spent on the impossible tasks (tDur), i.e. getting stuck in trying to solve something that is not solvable, indicating that long tDurs are related to low valence.

The feature importance tables vary across the models, but some features are important in all of them. For arousal, EDA is consistently high in all three models, which is expected, as EDA is a sensitive indicator of arousal [30]. Whereas TBB is associated with attention allocation, engagement, and emotional valence [42], which may explain why TBB is one of the most important features both in cognitive arousal and valence RF models. Harmful sisu S2 is high in all models as well, suggesting that tendency to stubbornness is associated with high arousal, possibly via tDur. Interestingly, the other stress biosignal RMSSD is not very important.

The data for **dominance** was imbalanced with only 18 samples for the low dominance class and 34 samples for the high dominance. Using the same settings for the models, each failed to detect the lower class. A similar result was seen in the classification of Cold tasks, in which all emotional dimensions are similarly imbalanced. For these results, please see Appendix B.

### 3.3 Dealing with imbalanced data

**Figure 7:**
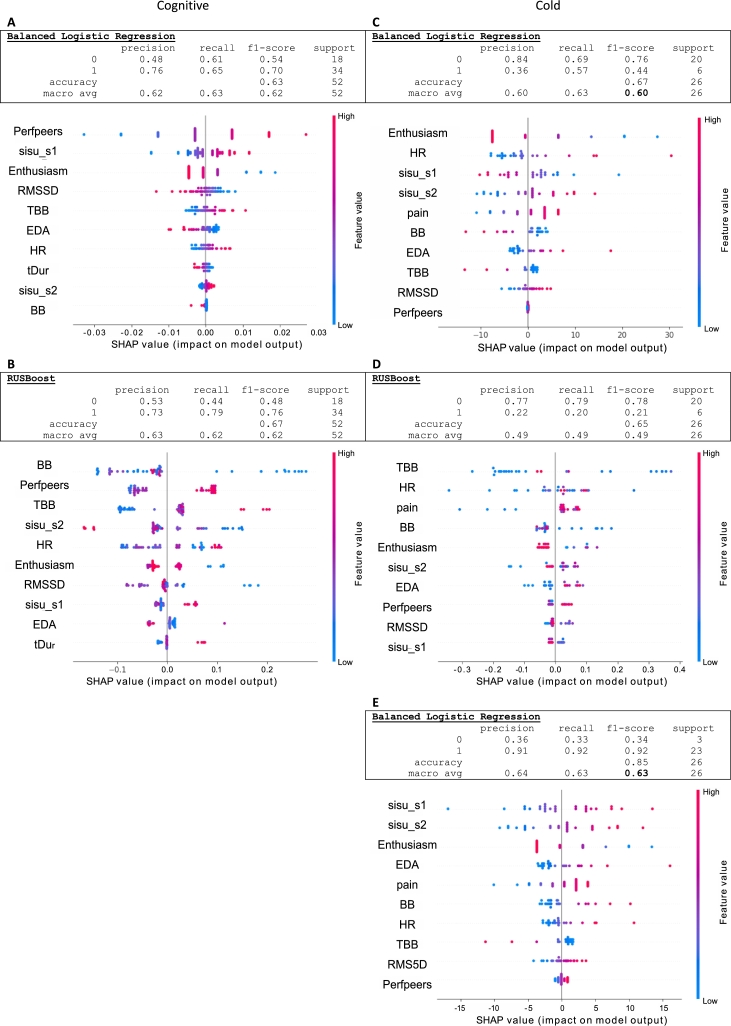
The best models for classification of Cognitive task dominance (A,B) and Cold task arousal (C,D) and valence (E). For these tasks the data were imbalanced, which is compensated in these models. The SHAP values indicate the importance of the feature in the model and SHAP values are colored to indicate the direction of the effect.

The problem at hand is not only a small data set but in some tasks, also a highly imbalanced one. Consequently, different techniques should be used in model selection and evaluation [77]. For instance, we can avoid the necessity of using nested CV by utilizing unoptimized models that do not require hyperparameters tuning (e.g., GNB and LR without regularization) or set the hyperparameters manually to some reasonable values (e.g., RF with a specified number of estimators) or alternatively using classifiers with an inner balancing sampler that balances the subsamples from the data set before performing the classification (e.g., RUSBoost classifier and Balanced AdaBoost classifier). We selected 1) Balanced logistic regression without regularizer, 2) Balanced Random Forest with 7 estimators, 3) Gaussian Naive Bayes, 4) Balanced AdaBoost, and 5) RusBoost, used them for cognitive task dominance and cold task classification, and evaluated these over 10 repeats of 3 stratified CV folds. Fig. 7 shows the best models arising from this test round for Cognitive dominance and Cold arousal and valence.

The best model with good accuracy, balanced low/high class recall and f1 was Balanced LR (Fig. 7 A, C, and E), even if the accuracy in RUSBoost was slightly better (Fig. 7 B). In Cognitive task dominance (Fig. 7 A and B), perfPeers is an important feature both in LR and RUSBoost, with positive association to dominance, but importance of the other features is quite different between the models. In RUSBoost, the distributions of SHAP values per feature are more clustered than in balanced LR. In Cold task arousal (Fig. 7 C and D), balanced LR is clearly the best model, both in terms of accuracy and recall. Enthusiasm and beneficial sisu S1 seem connected to low arousal, while HR, harmful sisu S2 and experienced pain are related to high arousal. It is noteworthy that even if the model (LR) is basically the same as in the classification of the cognitive task arousal, the list of important features is quite different. This probably reflects the nature of the task: as stressors, cognitive load, and physical pain stimulate different physiological processes and pathways, and their associations to personality and behavioral factors are different.

Care should be taken when interpreting the feature importance in models where a minority class has been largely misclassified, as the contribution from the minority class is negligible [46]. Clearly, the accuracy alone is not a reliable indicator of good performance in ill-balanced data sets, even when using algorithms that try to compensate the class balance: the model may demonstrate a reasonable accuracy but still neglect the less populated class, seen as heavily biased recall and f1 values. For the most imbalanced case Cold dominance, no unbalanced model was able to detect the low-populated class at all - even if the accuracy of the classification was very good (0.92 for each model). Using balanced algorithms did not much improve the recall: ADABoost had recall of 0.1 while all other models resulted in zero low-class recall, again with high value for accuracy. These unreliable results are not shown in Fig. 7 but can be seen in Appendix B. A similar trend was seen in Cold valence with a minority class of three samples (Fig. 7 E). Furthermore, the nested cross-validation often results in ill-defined metrics when splitting small, unbalanced data into 3 inner and 3 outer folds. In such cases, nested CV and hyperparameter tuning may not be a good option, and simpler models should be employed instead.

### 3.4 Discussion about ML results

The best-performing models for well-balanced Cognitive task data (arousal and valence, both with 52 samples) were both linear LR, GNB, and nonlinear (RF, KNN). For the Cold tasks (26 samples) and also the imbalanced Cognitive dominance, LR performed the best. This result matches the guidelines given in [77]: in small data sets, the models should be kept simple to avoid overfitting and improve the generalizability of the model. Therefore, we suggest using simple models and address the possible class imbalance.

Another direction would be to generate synthetic data to have larger dataset. However, the validation of synthetic data and measuring its authenticity is not trivial and needs more investigation. For example, we conducted supplementary experiments to explore alternative approaches to balance the classes. The known Synthetic Minority Over-sampling Technique (SMOTE) was used to generate synthetic samples. However, these experiments yielded unfavorable results and did not demonstrate any notable improvement in our specific case. Additionally, the balanced logistic regression outperformed the SMOTE. As such, we have chosen not to include these results in the presented findings. These additional experiments serve as an important reference point, highlighting the challenges and limitations encountered during generating synthetic samples. For instance, SMOTE generates synthetic samples based on existing minority class samples, there is a risk of overfitting, especially when the number of original minority class instances is small. The synthetic samples may overly represent the existing minority class instances, which can lead to the model being overly sensitive to those specific instances and performing poorly on unseen data. Therefore, future research should focus on the development of novel methods that can effectively address these limitations, providing more representative and diverse synthetic samples for imbalanced datasets. These new methods can further enhance the performance and generalizability of machine learning models trained on imbalanced data.

The linear models used in our study have certain requirements for the data. GNB has two assumptions: the input data are distributed normally and the features are independent. The distribution in a small data set is difficult to validate, but GNB has been used in similar data sets [74], [48], [29]. The features were selected to represent different aspects of the task experience and can be assumed to be reasonably independent. LR assumes independent, meaningful features and additionally binary outcomes, which all are satisfied.

There are a few other studies comparing ML methods in classification of emotions. Experienced fear levels, either on a binary or four-level scale, were classified in [2] based on signals from EEG and a variety of other biosignals. Using a k-fold CV (30 percent test samples). In this data set, derived from the DEAP database of 32 subjects [29], RF was found to perform best in terms of accuracy and F1. [2] also discusses the effects of feature pre-selection and in-model selection choices in detail. In a work on classification of anxiety, boredom, engagement, frustration, and anger, while solving anagrams and playing Pong [36], SVM demonstrated the best accuracy. The emotional states of the 15 participants, based on self-assessment, were classified into three levels for each emotion. However, on both of these papers, the classification tasks were designed in a different manner from our models: even if the ground truth is based on subjective appraisals, the classification was based on specific emotions rather than dimensions of the 3D emotional space.

The accuracies achieved in this work are similar to the ones reached in previous works (e.g., [18], [8]). However, evaluation of the model performance in such small data set is not trivial. Leave-one-out shows reasonable accuracy but introduces variation which can be overcome by using nested CV. In some cases, the models completely omit the minority class, and the classification may not generalize well [77]. Furthermore, the importance of accuracy, precision, and recall depends on the research question e.g. in detection of an unwanted state (such as cancer diagnostics or cognitive performance monitoring in safety-critical tasks), false negatives are intolerable while false positives can be allowed to a degree. Especially in such cases, accuracy may not be the best indicator of performance.

The number of features selected for ML was small with only one feature representing each biosignal source and few behavioral parameters. Often a large number of features is computed from e.g. biosignal data and used in the model. This results in a kind of black box where the association between results and feature importance is not very intuitive. There are contexts where this approach is optimal. However, we wanted to be able to see how the signal sources influence the classification. Also, some of the models we used assume independent features (GNB, LR). [36] discusses the importance of feature pre-selection and even proposes that for each individual, only “useful” features (i.e. responses correlating with the subjectively assessed emotional state) should be used, and these may vary across the participants. Another choice we made was to construct the feature set from biosignals, sisu scores, and task performance parameters; typically only biosignals and other monitoring data are used. This mix was constructed to investigate the role of these in the classification results. Adding personality features and/or subjective annotations is a way to improve specificity when optimizing (teaching) ML-based stress and emotion detection solutions in real-life context. For instance, in predicting cognitive valence PrefPeers (behavioral feature) emerged as the most important feature, similarly, when predicting cold valence Sisu scores (personality feature) were more important than biosignals features. Hence, we recommend to include features representing physiology, behavior and personality.

SHAP feature importance plots provide an easy, model-independent way to see the role of different features in the classification. However, in cases where the model performance, either in terms of accuracy or precision/recall, especially with imbalanced data sets, is compromised, also the SHAP plots should be considered critically. The predictions generated by different models can differ based on the inherent assumptions and biases of each model, and the way they are interpreted can also vary among the models. For instance, a linear model and a tree model could have comparable levels of accuracy, but they represent different relationships between variables.

## 4 Conclusions

This paper presents a novel protocol to induce different mental states in realistic yet controlled stress conditions, combined with comprehensive biosignal monitoring. Physiological features, task performance data, and sisu personality traits were used to classify the subjectively reported post-task emotional state: valence, arousal and dominance. Five common ML methods were compared in the binary classification of the three emotional state dimensions, assessed after the task and divided into high and low-intensity classes. The best models varied from one dataset to another but for all the cases, simple linear models performed well, especially for small datasets. In addition to features derived from psychophysiological signals, behavioral and personality features contribute to the classification of emotional states. Compared to LOOCV, nested CV decreased the variance between the CV loops.

The conclusions from our experiments for interpretable classification of human mental state from biosignals and behavioral data, typically with low number of samples and high inter-individual variability, can be summarized: if possible, include features representing physiology, behavior and personality, use simple models, address the possible class imbalance, and use stratified nested cross-validation. How well these learnings generalize to different setups and research questions remains to be seen. Nevertheless, in this evolving branch of affective computing, more work systematically comparing available methods is most welcome.

## CRediT authorship contribution statement

**Heba Sourkatti:** Methodology, Software, Validation, Visualization, Writing – original draft, Writing – review & editing. **Kati Pettersson:** Conceptualization, Funding acquisition, Validation, Writing – original draft, Writing – review & editing. **Bart van der Sanden:** Data curation, Formal analysis, Software. **Mikko Lindholm:** Data curation. **Johan Plomp:** Conceptualization. **Ilmari Määttänen:** Conceptualization, Methodology, Resources. **Pentti Henttonen:** Methodology, Resources, Software. **Johanna Närväinen:** Conceptualization, Funding acquisition, Investigation, Methodology, Project administration, Supervision, Writing – review & editing.

## Declaration of Competing Interest

The authors declare that they have no known competing financial interests or personal relationships that could have appeared to influence the work reported in this paper.

## Figures and Tables

**Table A.6:** Cognitive task, post-task arousal.

**Model**	**Parameters**
**LR**	C: 0.0215
**RF**	criterion: ‘gini’, max_depth: 5, min_samples_split: 2, n_estimators: 20
**SVM**	C: 0.1, kernel: ‘linear’
**KNN**	n_neighbors: 3
**GNB**	none

**Table A.7:** Cold task, post-task arousal.

**Model**	**Parameters**
**LR**	C: 1000
**RF**	criterion: ‘gini’, max_depth: 5, min_samples_split: 2, n_estimators: 5
**SVM**	C: 2.154, kernel: ‘rbf’
**KNN**	n_neighbors: 4
**GNB**	none

**Table A.8:** Cognitive task, post-task emotions.

**Model**	**Parameters**
*Arousal*	
**LR**	C: 0.02154
**RF**	criterion: ‘gini’, max_depth: 5, min_samples_split: 2, n_estimators: 20
**SVM**	C: 0.1, kernel: ‘linear’
**KNN**	n_neighbors: 5
**GNB**	none

*Valence*	
**LR**	C: 0.0215
**RF**	criterion: ‘gini’, max_depth: 15, min_samples_split: 9, n_estimators: 10
**SVM**	C: 0.0215, kernel: ‘linear’
**KNN**	n_neighbors: 4
**GNB**	none

*Dominance*	
**LR**	C: 0.1
**RF**	criterion: ‘gini’, max_depth: 10, min_samples_split: 2, n_estimators: 20
**SVM**	C: 10.0, kernel: ‘linear’
**KNN**	n_neighbors: 5
**GNB**	none

**Table A.9:** Cold task, post-task emotions.

**Model**	**Parameters**
*Arousal*	
**LR**	C: 10.0
**RF**	criterion: ‘gini’, max_depth: 5, min_samples_split: 9, n_estimators: 5
**SVM**	C: 0.1, kernel: ‘rbf’
**KNN**	n_neighbors: 4
**GNB**	none

*Valence*	
**LR**	C: 46.42
**RF**	criterion: ‘gini’, max_depth: 5, min_samples_split: 2, n_estimators: 20
**SVM**	C: 0.001, kernel: ‘rbf’
**KNN**	n_neighbors: 4
**GNB**	none

*Dominance*	
**LR**	C: 0.001
**RF**	criterion: ‘gini’, max_depth: 5, min_samples_split: 2, n_estimators: 5
**SVM**	C: 2.154, kernel: ‘rbf’
**KNN**	n_neighbors: 3
**GNB**	none

**Figure B.8:**
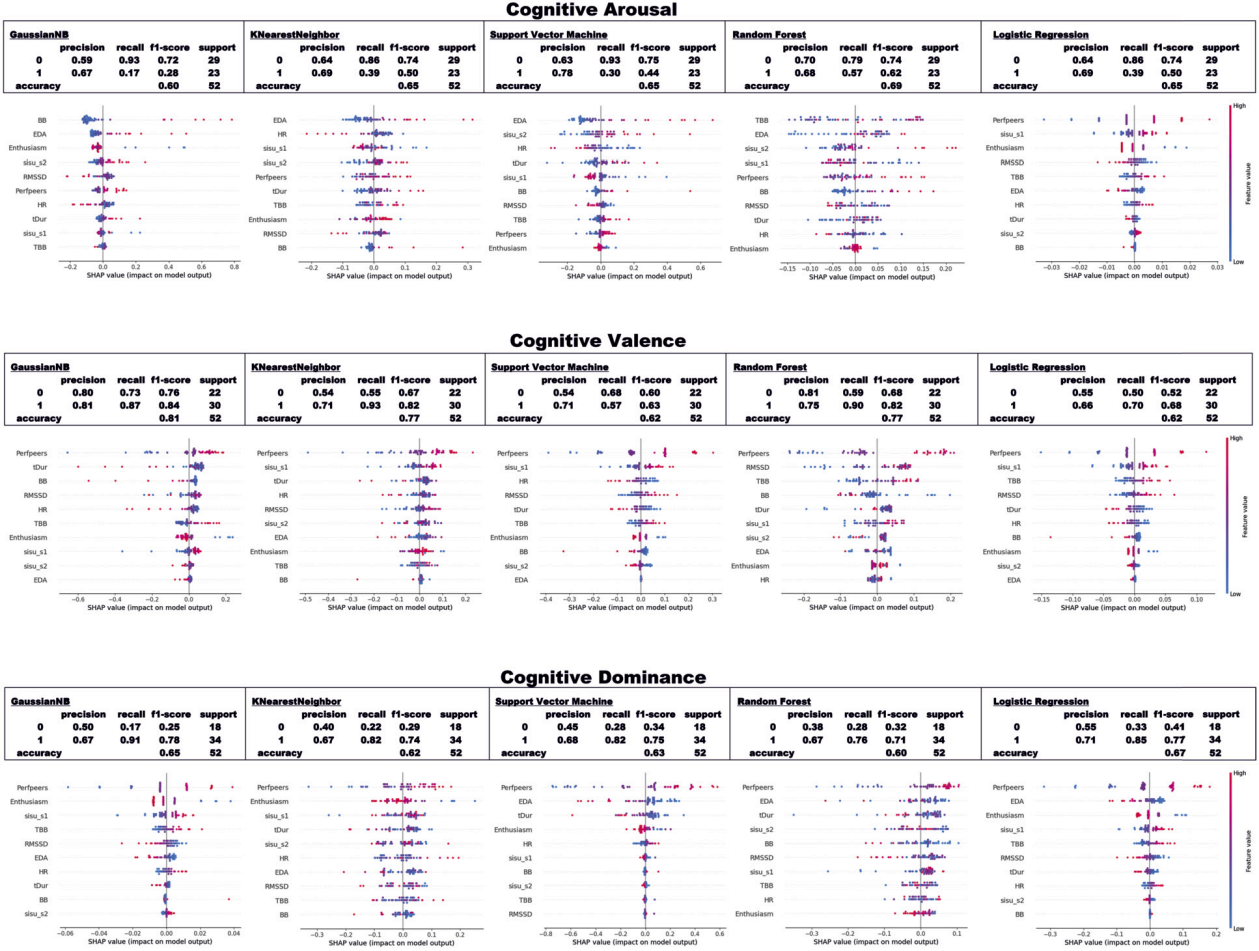
Full report of classification of arousal, valence and dominance in the cognitive task.

**Figure B.9:**
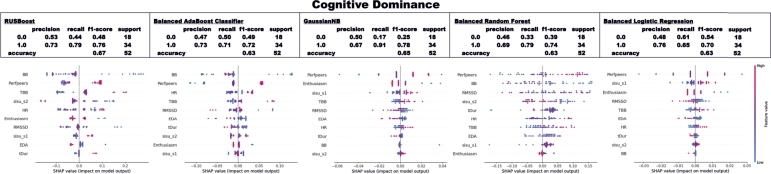
Full report of balanced classification of dominance in the cognitive task.

**Figure B.10:**
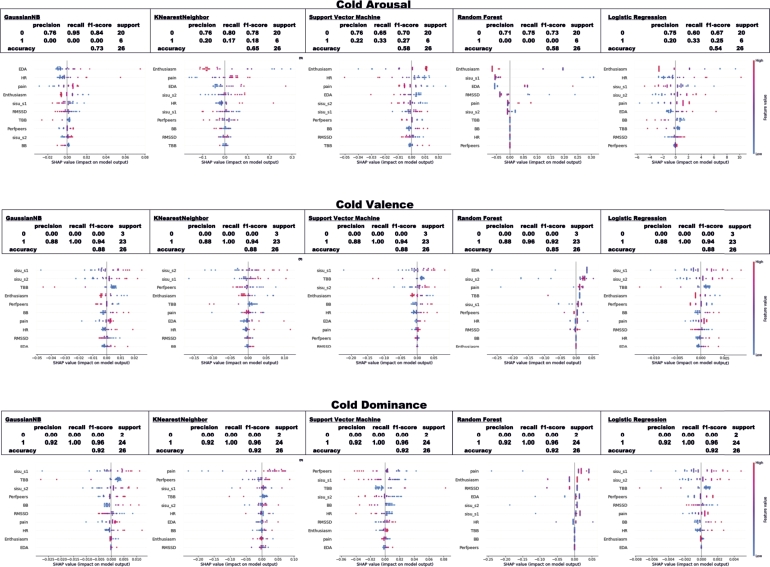
Full report of classification of arousal, valence and dominance in the cold pressor task.

**Figure B.11:**
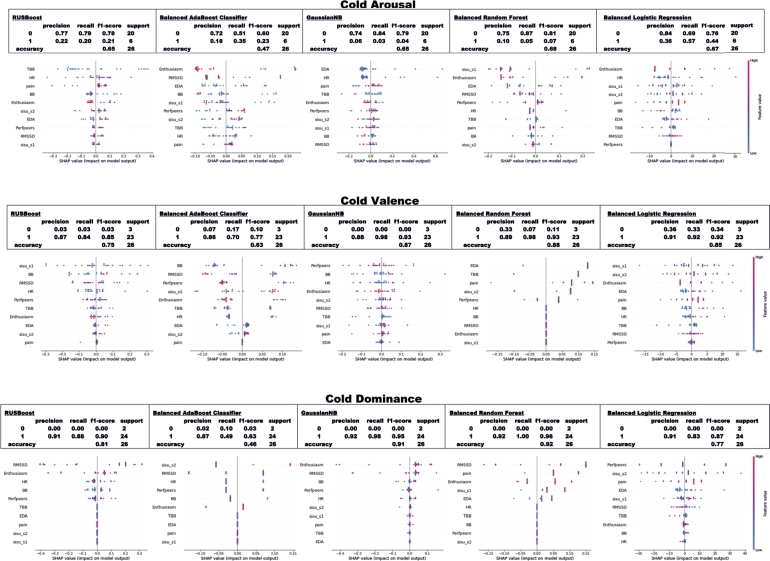
Full report of balanced classification of arousal, valence and dominance in the cold pressor task.

## Data Availability

The data associated with the study has not been deposited into a publicly available repository as the authors do not have permission to share the data.

## Appendix A Model parameters and hyperparameters

• Linear Regression LR: C to control the regularization, where higher values reduce the regularization.
• Random Forest RF: number of estimator, criterion (gini or entropy), maximum depth, minimum samples split
• Support Vector Machine SVM: kernel (linear or rbf), regularization parameter C
• K Nearest Neighbors KNN: number of neighbors
• Gaussian Naive Bayes GNB: none
• For all models: random_state = 7

### A.1 Leave-one-out CV predictions for post-task arousal after cognitive and cold tasks

See Table A.6, Table A.7.

### A.2 Nested CV predictions for post-task arousal, valence and dominance after cognitive task

See Table A.8, Table A.9.

## Appendix B Full figure panels of results

See Figure B.8, Figure B.9, Figure B.10, Figure B.11.

## Appendix C Supplementary material

Supplementary material 1 Research information document provided to participant before signing the consent. Supplementary material 1

Supplementary material 2 Consent document for participating in the research. Supplementary material 2
