# Supplementary material for: Investigation of different ML approaches in classification of emotions induced by acute stress
Source: Heliyon. 2023 Dec 11;10(1):e23611. doi: 10.1016/j.heliyon.2023.e23611 (PMC10761802; doi:10.1016/j.heliyon.2023.e23611)
Supplement: Supplementary material 1 — Research information document provided to participant before signing the consent. [file mmc1.docx]

**Annan suostumukseni seuraavaan:**

1 Olen lukenut ja ymmärtänyt tutkimustiedotteessa tutkimuksesta annetut tiedot. Antamieni ja mitattujen tietojen käyttö, luottamuksellisuus ja säilytys on selostettu minulle. Minulle on annettu riittävä mahdollisuus esittää kysymyksiä tutkimuksen toteutuksesta ja tutkittavan oikeuksista.

kyllä ⃞ ei ⃞

2. Ymmärrän, että koetilanne sisältää stressaavia ja/tai fyysistä epämukavuutta aiheuttavia osioita. Vakuutan, että terveydentilastani ja muista kokeeseen osallistumiseen vaikuttavista tekijöistä antamani tiedot ovat totuudenmukaisia. En osallistu mittauksiin sairaana tai väsyneenä.

kyllä ⃞ ei ⃞

3. Osallistun vapaaehtoisesti tutkimukseen. Suostun osallistumaan koetilanteisiin annettujen ohjeiden mukaisesti. Ymmärrän, että voin milloin tahansa keskeyttää tai jättää välistä mitä tahansa sen alaosioista syytä ilmoittamatta.

kyllä ⃞ ei ⃞

4. Olen tietoinen oikeudestani peruuttaa suostumukseni minusta kerätyn tutkimusaineiston käyttöön ilmoittamalla siitä tutkijoille milloin tahansa, jolloin kaikki minusta kerätyt tiedot poistetaan.

kyllä ⃞ ei ⃞

5 Ymmärrän, että tutkimuksen tekijöillä ja muilla tutkijoilla on pääsy kerättyihin tietoihin vain, jos he ovat sitoutuneet tiedon luottamukselliseen käsittelyyn. Tutkimustuloksiani saa käyttää tieteelliseen raportointiin sellaisessa muodossa, jossa yksittäistä tutkittavaa ei voi tunnistaa.

kyllä ⃞⃞

**Osallistuja:**

________________________ ____________________________ ______________

Nimi Allekirjoitus Päiväys

**Lomakkeen vastaanottava tutkija:**

________________________ ____________________________ ______________

Nimi Allekirjoitus Päiväys

Vastaavat tutkijat:

Helsinki:

Ilmari Määttänen, Helsingin Yliopisto

[ilmari.maattanen@helsinki.fi](mailto:ilmari.maattanen@helsinki.fi)

puh. 050 518 4083

Kuopio:

Johanna Närväinen, VTT Kuopio

[johanna.narvainen@vtt.fi](mailto:johanna.narvainen@vtt.fi)

puh. 040 6747905
