# Supplementary material for: Investigation of different ML approaches in classification of emotions induced by acute stress
Source: Heliyon. 2023 Dec 11;10(1):e23611. doi: 10.1016/j.heliyon.2023.e23611 (PMC10761802; doi:10.1016/j.heliyon.2023.e23611)
Supplement: Supplementary material 2 — Consent document for participating in the research. [file mmc2.docx]

Tutkimustiedote: Henkiset voimavarat-tutkimus (Kuopio)

Kiitos tutkimustamme kohtaan osoittamastasi kiinnostuksesta! Teknologian tutkimuskeskus VTT mukana Suomen Akatemian rahoittamassa hankkeessa (313399), jonka osana tutkitaan henkisten voimavarojen ilmenemistä laboratorio-oloissa kokeellisissa stressitilanteissa.

Tutkimuksessa selvitetään käyttäytymistä ja kehon toimintaa ja erilaisissa keskittymistä ja epämukavuuden sietoa vaativissa stressitilanteissa, esimerkiksi yksinkertaisilla laskutehtävillä tai lämpötilan vaihteluilla. Osa tehtävistä aiheuttaa fyysistä epämukavuutta. Ennen tutkimusta täytetään taustatietokysely, jossa kysytään henkisten voimavarojen lisäksi yleisiä terveydentilaasi kuvaavia kysymyksiä.

Kaikki tehtävät kestävät maksimissaan 5 minuuttia, ovat turvallisia ja jokainen suorittaa niitä juuri sen mukaan, mikä tuntuu itselle sopivalta. Tehtävät esitellään ennen niiden suorittamista, ja voit päättää jokaisen kohdalla, haluatko osallistua siihen. Ennen ja jälkeen jokaista tehtävää arvioit sitä ja suoriutumistasi suhteessa itseesi ja muihin ihmisiin. Kokeen jälkeen saat kattavan palautteen suoriutumisestasi.

Tutkimuskäynnin kesto on noin kaksi tuntia, josta mittalaitteiden kiinnitys kestää noin tunnin. Koetilanteessa mittaamme kehon ja aivojen reaktiota erilaisilla mittareilla. Mitattavat signaalit ovat ihon sähkönjohtavuus kädessä, syke (kylkeen ja kaulan alapuolelle sijoitettavat elektrodit), kasvolihasaktivaatio (kurtistus- ja hymylihakset) ja aivosähkökäyrä EEG. Mittalaitteet ovat turvallisia ja täysin haitattomia, eivätkä edellytä sinulta erityisiä vaatimuksia kokeen aikana. Ihonsähkönjohtavuus, pulssi ja hengitystaajuus kertovat yleisestä vireystilastasi, kasvojen lihasten toiminta yleisestä tunnetilastasi ja EEG erilaisista aivotoimintaan liittyvistä tiloista, kuten esim. tarkkaavaisuus ja kiinnostus. Keholle elektrodit kiinnitetään liimatarralla, joka on helppo ottaa pois.

EEG-mittauksessa käytetään vedellä pois pestävää elektrolyyttigeeliä. Tarjoamme mahdollisuuden hiusten pesuun mittauksen jälkeen.

Tutkimusavustaja kiinnittää mittalaitteet yksityisessä valmistelutilassa, johon on pääsy vain tutkimuksen suorittajilla. Itse koe tehdään erillisessä huoneessa, johon on käynti valmistelutilasta. Tutkimukseen osallistuminen on vapaaehtoista ja luottamuksellista. Ennen koetta allekirjoitetaan suostumuslomake. Tutkimustulokset säilytetään rekisterissä arkistossa, josta poistetaan henkilökohtaiset tunniste- ja yksilöintitiedot. Rekisteriseloste on nähtävillä pyynnöstä.

Tutkimuksen voi keskeyttää missä tahansa vaiheessa. Suostumuksen kerättyjen tutkimustietojen käyttöön voi peruuttaa milloin vain, jolloin kaikki siihen mennessä kerätyt tiedot poistetaan. Kerätyn aineiston pohjalta saadut tutkimustulokset julkaistaan vertaisarvioiduissa tieteellisissä aikakauslehdissä.

Etsimme tutkimukseen täysi-ikäisiä, perusterveitä, oikeakätisiä henkilöitä. Tutkimukseen ei voi osallistua, jos kärsit kroonisesta sydänsairaudesta, tyypin I diabeteksesta, lääkitystä vaativasta masennuksesta, tai mikäli käytät keskushermostoon vaikuttavia lääkkeitä. Tutkimukseen ei voi tulla sairaana tai poikkeuksellisen väsyneenä. Tutkimusaika sovitaan erikseen.

Korvaukseksi ajankäytöstä ja vaivannäöstä saat kaksi Finnkinon elokuvalippua.

Tutkimus tehdään

Technopoliksessa Microkatu 1, Q-siipi, 2. krs.

Tutkimuksen vastaava tutkija on:

Johanna Närväinen, VTT Kuopio, VTT PL 1199, 70211 Kuopio

johanna.narvainen@vtt.fi

puh 040 6747905
